# Observing the Observer (II): Deciding When to Decide

**DOI:** 10.1371/journal.pone.0015555

**Published:** 2010-12-14

**Authors:** Jean Daunizeau, Hanneke E. M. den Ouden, Matthias Pessiglione, Stefan J. Kiebel, Karl J. Friston, Klaas E. Stephan

**Affiliations:** 1 Wellcome Trust Centre for Neuroimaging, University College of London, London, United Kingdom; 2 Brain and Spine Institute, Hôpital Pitié-Salpêtrière, Paris, France; 3 Laboratory for Social and Neural Systems Research, Institute of Empirical Research in Economics, University of Zurich, Zurich, Switzerland; 4 Max Planck Institute for Human Cognitive and Brain Sciences, Leipzig, Germany; 5 Donders Institute for Brain, Cognition and Behaviour, Nijmegen, The Netherlands; Indiana University, United States of America

## Abstract

In a companion paper [1], we have presented a generic approach for inferring how subjects make optimal decisions under uncertainty. From a Bayesian decision theoretic perspective, uncertain representations correspond to “posterior” beliefs, which result from integrating (sensory) information with subjective “prior” beliefs. Preferences and goals are encoded through a “loss” (or “utility”) function, which measures the cost incurred by making any admissible decision for any given (hidden or unknown) state of the world. By assuming that subjects make optimal decisions on the basis of updated (posterior) beliefs and utility (loss) functions, one can evaluate the likelihood of observed behaviour. In this paper, we describe a concrete implementation of this *meta-Bayesian* approach (i.e. a Bayesian treatment of Bayesian decision theoretic predictions) and demonstrate its utility by applying it to both simulated and empirical reaction time data from an associative learning task. Here, inter-trial variability in reaction times is modelled as reflecting the dynamics of the subjects' internal recognition process, i.e. the updating of representations (posterior densities) of hidden states over trials while subjects learn probabilistic audio-visual associations. We use this paradigm to demonstrate that our meta-Bayesian framework allows for (i) probabilistic inference on the dynamics of the subject's representation of environmental states, and for (ii) model selection to disambiguate between alternative preferences (loss functions) human subjects could employ when dealing with trade-offs, such as between speed and accuracy. Finally, we illustrate how our approach can be used to quantify subjective beliefs and preferences that underlie inter-individual differences in behaviour.

## Introduction

How can we infer subjects' beliefs and preferences from their observed decisions? Or in other terms, can we identify the internal mechanisms that led subjects to act, as a response to experimentally controlled stimuli? Numerous experimental and theoretical studies imply that subjective prior beliefs, acquired over previous experience, strongly impact on perception, learning and decision-making ([2]–[6]). We also know that preferences and goals can impact subjects' decisions in a fashion which is highly context-dependent and which subjects may be unaware of ([7]–[8]). But how can we estimate and disentangle the relative contributions of these components to observed behaviour? This is the nature of the so-called Inverse Bayesian Decision Theory (IBDT) problem, which has been a difficult challenge for analytical treatments.

In a companion paper [1], we have described a variational Bayesian framework for approximating the solution to the IBDT problem in the context of perception, learning and decision-making studies. Subjects are assumed to act as Bayesian observers, whose recognition of the hidden causes of their sensory inputs depends on the inversion of a *perceptual model* with subject-specific priors. The Bayesian inversion of this perceptual model derives from a variational formulation, through the minimization of sensory surprise (in a statistical sense). More precisely, the variational Bayesian approach minimizes the so-called “free energy”, which is a lower bound on (statistical) surprise about the sensory inputs. The ensuing probabilistic subjective representation of hidden states (the posterior belief) then enters a *response model* of measured behavioural responses. Critically, decisions are thought to minimize expected loss or risk, given the posterior belief and the subject-specific loss (or utility) function that encodes the subject's preferences. The response model thus provides a complete mechanistic mapping from experimental stimuli to observed behaviour. Over time or trials, the response model has the form of a state-space model (e.g., [9]), with two components: (i) an evolution function that models perception and learning through surprise minimization and (ii) an observation function that models decision making through risk minimization.

Solving the IBDT problem, or *observing the observer*, then reduces to inverting this state-space response model, given experimentally measured behaviour. This meta-Bayesian approach (experimenters make Bayesian inferences about subject's Bayesian inferences) provides an approximate solution to the IBDT problem in that it enables comparisons of competing (perceptual and response) models and inferences on the parameters of those models. This is important, since evaluating the evidence of, for example, different response models in the light of behavioural responses means we can distinguish between different loss functions (and thus preferences) subjects might have.

This paper complements the theoretical account in the companion paper by demonstrating the practical applicability of our framework. Here, we use it to investigate what computational mechanisms operate during learning-induced motor facilitation. While it has often been found that (correct) expectations about sensory stimuli speed up responses to those stimuli (e.g. [10]–[11]), explaining this acceleration of reaction times in computationally mechanistic terms is *not* trivial. We argue that such an explanation must take into account the dynamics of subjective representations, such as posterior beliefs about the causes that generate stimuli, and their uncertainty, as learning unfolds over trials. Throughout the text, “representation” refers to posterior densities of states or parameters. We investigate these issues in the context of an audio-visual associative learning task [12], where subjects have to categorize visual stimuli as quickly as possible. We use this task as a paradigmatic example of what sort of statistical inference our model-based approach can provide. As explained in detail below, this task poses two interesting explananda for computational approaches: (i) it relies upon a hierarchical structure of causes in the world: visual stimuli depend probabilistically on preceding auditory cues whose predictive properties change over time (i.e., a volatile environment), and (ii) it introduces a conflict in decision making, i.e. a speed-accuracy trade-off.

We construct two Bayesian decision theoretic (BDT) response models based upon the same speed-accuracy trade-off (c.f. [13] or [14]), but differing in their underlying perceptual model. These two perceptual models induce different learning rules, and thus different predictions, leading to qualitatively different trial-by-trial variations in reaction times. We have chosen to focus on reaction time data to highlight the important role of the response model and to show that optimal responses are not just limited to categorical choices.

Of course, the validity of a model cannot be fully established by application to empirical data whose underlying mechanisms or “ground truth” are never known with certainty. However, by ensuring that only one of the competing models was fully consistent with the information given to the subjects, we established a reference point against which our model selection results could be compared, allowing us to assess the construct validity of our approach. Furthermore, we also performed a simulation study, assessing the veracity of parameter estimation and model comparison using synthetic data for which the ground truth was known.

## Methods

How does learning modulate reaction times? In this section, we first describe the associative learning task, and then the perceptual and response models we have derived to model the reaction time data. We then recall briefly the elements of the variational Bayesian framework which is described in the companion paper in detail and which we use to invert the response model given reaction time data. Next, we describe the Monte-Carlo simulation series we have performed to demonstrate the validity of the approach. Finally, we summarize the analysis of real reaction time data, illustrating the sort of inference that can be derived from the scheme, and establishing the construct validity of the approach.

### The associative learning task

The experimental data and procedures have been reported previously as part of a functional magnetic resonance imaging study of audio-visual associative learning [12]. We briefly summarize the main points. Healthy volunteers were presented visual stimuli (faces or houses) following an auditory cue. The subjects performed a speeded discrimination task on the visual stimuli. On each trial, one of two possible auditory cues was presented (simple tones of different frequencies; C_1_ and C_2_), each predicting the subsequent visual cue with a different probability. The subjects were told that the relationship between auditory and visual stimuli was probabilistic and would change over time but that these changes were random and not related to any underlying rule. The reaction-time (from onset of visual cue to button press) was measured on each trial.

The probability of a given visual outcome or response cue, say *face*, given C_1_ was always the same as the probability of the alternative (*house*) given C_2_: 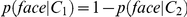. Moreover, since the two auditory cues occurred with equal frequency, the marginal probability of a face (or house) on any given trial was always 50%. This ensured that subjects could not be biased by *a priori* expectations about the outcome. In the original regression analyses in [12] no differences were found between high and low tone cues, nor any interactions between cue type and other experimental factors; here, we therefore consider the trials cued by C1 and C2 as two separate (intermingled, but non-interacting) sequences. This allows us to treat the two sequences as replications of the experiment, under two different auditory cues. We hoped to see that the results were consistent under the high and low tone cues.

A critical manipulation of the experiment was that the probabilistic cue-outcome association pseudorandomly varied over blocks of trials, from strong 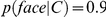, and moderate 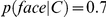, to random 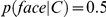. Our subjects were informed about the existence of this volatility without specifying the structure of these changes (timing and probability levels). We prevented any explicit search for systematic relationships by varying the length of the blocks and by presenting predictive and random blocks in alternation. In one session, each block lasted for 28–40 trials, within which the order of auditory cues was randomized. Each of five sessions lasted approximately seven minutes. On each trial, an auditory cue was presented for 300 ms, followed by a brief (150 ms) presentation of the visual outcome. In order to prevent anticipatory responses or guesses, both the inter-trial interval (2000±650 ms) and visual stimulus onset latency (150±50 ms) were jittered randomly.

The conventional analysis of variance (ANOVA) of the behavioural measures presented in [12] demonstrated that subjects learned the cue-outcome association: reaction times to the visual stimuli decreased significantly with increasing predictive strengths of the auditory cues. In what follows, we try to better understand the nature of this learning and the implicit perceptual models the subjects were using.

### Perceptual and response models

The first step is to define the candidate response models that we wish to consider. In what follows, we will restrict ourselves to two qualitatively different perceptual models, which rest on different prior beliefs and lead to different learning rules (i.e. posterior belief update rules or recognition processes). To establish the validity of our meta-Bayesian framework, the two models used for the analysis of the empirical data were deliberately chosen such that one of them was considerably less plausible than the other: whereas a “dynamic” model exploited the information given to the subjects about the task, the other (“static”) model ignored this information. This established a reference point for our model comparisons (akin to the “ground truth” scenario used for validating models by simulated data). These perceptual models were combined with a loss-function embodying the task instructions to form a complete BDT response model. This loss-function had two opposing terms, representing categorization errors and the decision time, respectively, and thus inducing the speed-accuracy trade-off of the task. We now describe the form of these probabilistic models and their inversion.

#### Perceptual models

**Figure 1:**
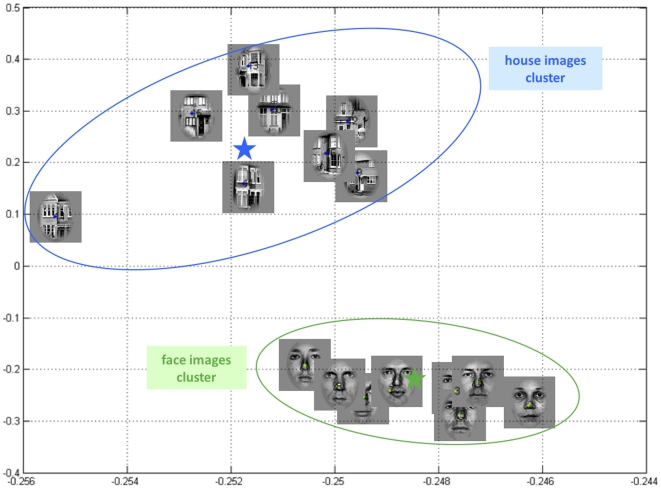
2D projection of the visual stimuli that were presented to the subjects (two sets of eight face images and eight house images, respectively). X-axis: first principal component, y-axis: second principal component. On this 2D projection, house and face images clearly cluster (green and blue ellipses) around “average” face and house (green and blue stars), respectively. One might argue that these ellipses approximate the relative ranges of variations of faces and houses, as perceived by the visual system.

The sensory signals (visual outcomes) 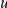 presented to the subjects were random samples from two sets of images, composed of eight different faces and eight different houses, respectively. A two-dimensional projection of these images onto their two first principal eigenvectors clearly shows how faces and houses cluster around two centres that can be thought of as an “average” face and house, respectively (see Figure 1). We therefore assumed the sensory inputs 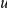 to be as a univariate variable (following some appropriate dimension reduction), whose expectation depends upon the hidden state (*face* or *house*). This can be expressed as a likelihood that is a mixture of Gaussians:
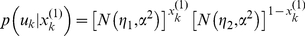
(1)


Here 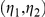 are the expected sensory signals caused by houses and faces (the “average” face and house images), 
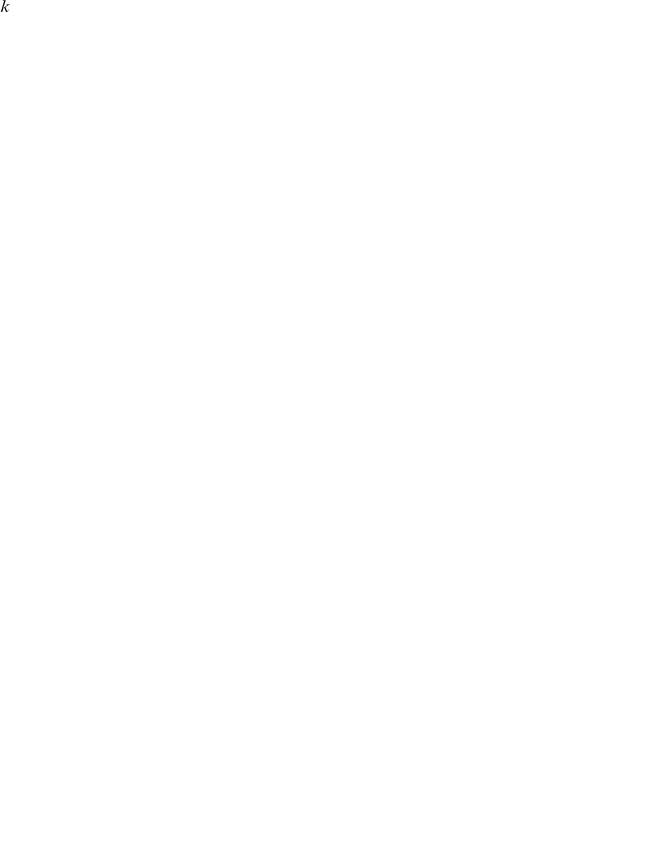
 is a trial index, 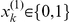 is an indicator state that signals the category (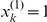: *house*, 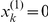: *face*), and 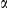 is the standard deviation of visual outcomes around the average face/house images. During perceptual categorization, subjects have to recognize 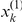, given all the sensory information to date. As faces and houses are well-known objects for whose categorisation subjects have a life-long experience, it is reasonable to assume that 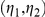 and 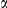 are known to the subjects. The hidden category states 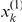 have a prior Bernoulli distribution conditioned on the cue-outcome associative strength 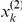:
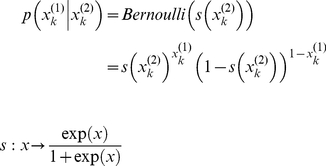
(2)


**Figure 2:**
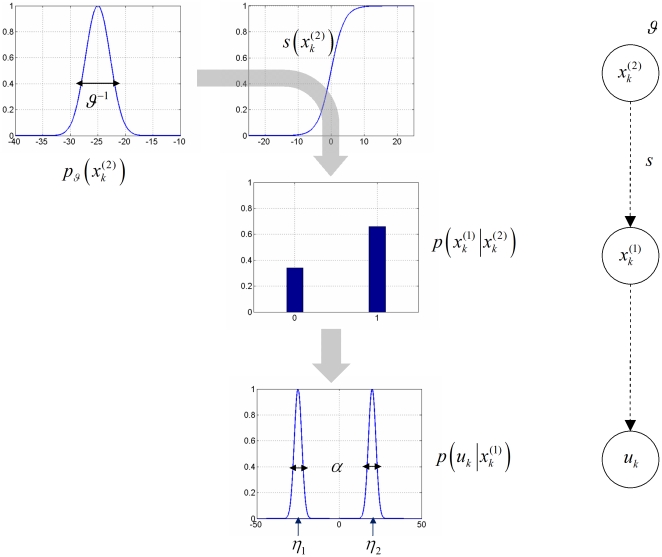
Conditional dependencies in perceptual models of associative learning. **Left**: cascade of events leading to the sensory outcomes. A Gaussian prior (with variance 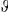) is defined at the level of the cue-outcome association 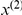. Passed through a sigmoid mapping, this determines the probability of getting a house (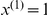) or a face (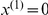). Finally, this determines the visual outcome 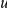 within the natural range of variation (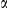) of house/face images. **Right**: Equivalent graphical model.

The sigmoid function 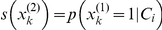 maps the associative strength 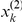 to the probability of seeing a house given the present auditory cue 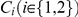. Figure 2 summarises the general structure of the perceptual models of associative learning in this paper.

We considered two perceptual models that differed only in terms of prior beliefs about the associative strength. Although both models have a prior expectation of zero for the associative strength, they differ profoundly in their predictions about how that associative strength changes over time. This is reflected by the different roles of the perceptual parameter 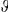 in the two models:

- The *static perceptual model*, 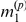: Subjects were assumed to ignore the possibility of changes in associative strength and treat it as stationary. Under this model, subjects assume that the associative strength has a constant value, 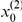, across trials and is sampled from a Gaussian prior; *i.e*.: 
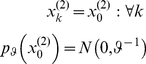
(3)where 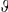 is its (fixed) prior precision. Here, the perceptual parameter 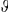 effectively acts as an (unknown) initial condition for the state-space formulation of the problem (see Equation 13 below).
- The *dynamic perceptual model* 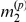: Subjects assumed *a priori* that the associative strength 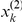 varied smoothly over time, according to a first-order Markov process. This is modelled as a random walk with a Gaussian transition density: 
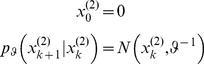
(4) Here, 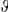 is the precision hyperparameter which represents the roughness (inverse smoothness) of changes in associative strength (*i.e.*, its volatility).

Note that the task information given to subjects did highlight the possibility of changes in cue strength. Therefore, from the point of view of the experimenter, it is more likely that the subjects relied upon the dynamic model to form their prior predictions. The choice of these two models was deliberate as it allowed for a clear prediction: we hoped to see that model comparison would show a pronounced superiority of the dynamic model (see section ‘Inverting the response model below’).

#### Recognition: the variational Bayesian inversion of the perceptual model

Given the perceptual models described above, we can now specify the recognition process in terms of their variational Bayesian inversion. The generic derivation of the recognition process is detailed in the companion paper [1]. In brief, subjects update their belief on-line, using successive stimuli to optimise 
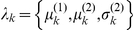
, the sufficient statistics of the posterior density on the k-*th* trial. Under a mean-field/Laplace approximation to the joint posterior, these sufficient statistics are (i) 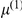, the first-order moment of the Bernoulli posterior 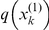 about the outcome category 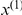, and (ii) 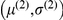, the first- and second- order moments of the Gaussian posterior 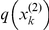 about the associative strength 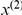. The recognition process derives from the minimization of the surprise conveyed by sensory stimuli at each trial. Within a variational Bayesian framework, negative surprise is measured (or, more precisely, lower-bounded) via the so-called perceptual free-energy 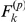 [15]:
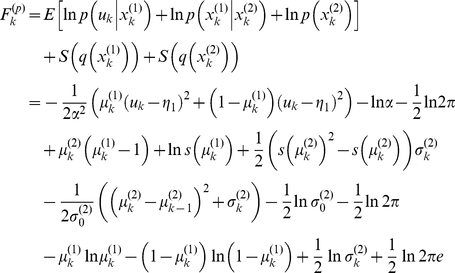
(5)where the expectation is taken under the approximate posterior densities (representations) 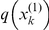 and 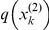 and 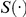 denotes the Shannon entropy. Note that the variance parameter 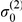 depends on the perceptual model; i.e.
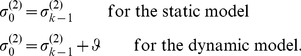
(6)


Note also that the perceptual free energy 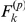 of the 
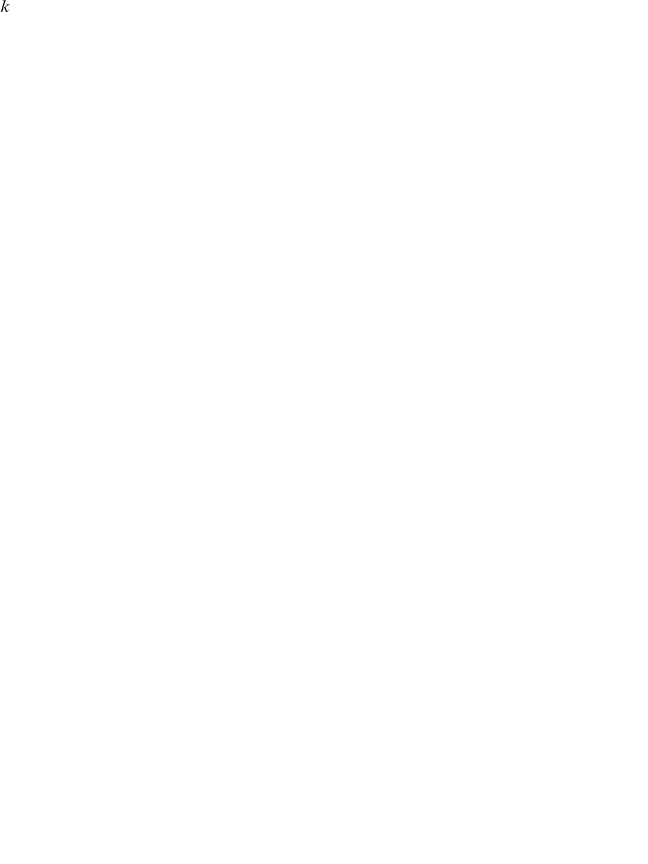
-th trial depends on the representation of associative strength at the previous trial, through the sufficient statistics 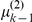 and 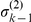. Therefore, these affect the current optimal sufficient statistics 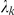 (including that of the outcome category), allowing learning to be expressed over trials. Optimizing the perceptual free energy 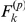 with respect to 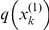 and 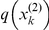 yields the updated posterior densities of both the outcome category (*face* or *house*)
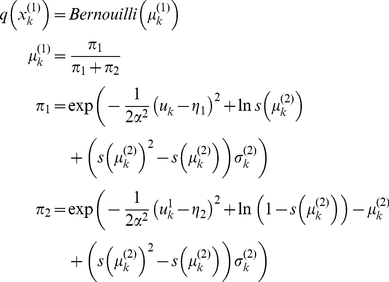
(7)and of the associative strength
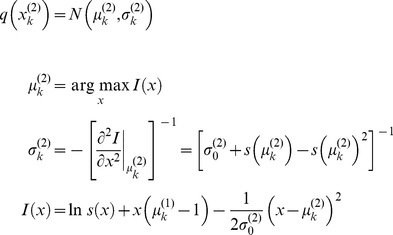
(8)


Note that functional form of the sufficient statistics above depends upon the perceptual model, through the variance parameter 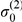, which in turn depends upon the precision parameter 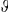 (see Equation 6). This dependence is important, since it strongly affects the recognition process. Under the static perceptual model, equation 8 tells us that the subject's posterior variance 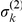 about the associative strength is a monotonically decreasing function of trial index 
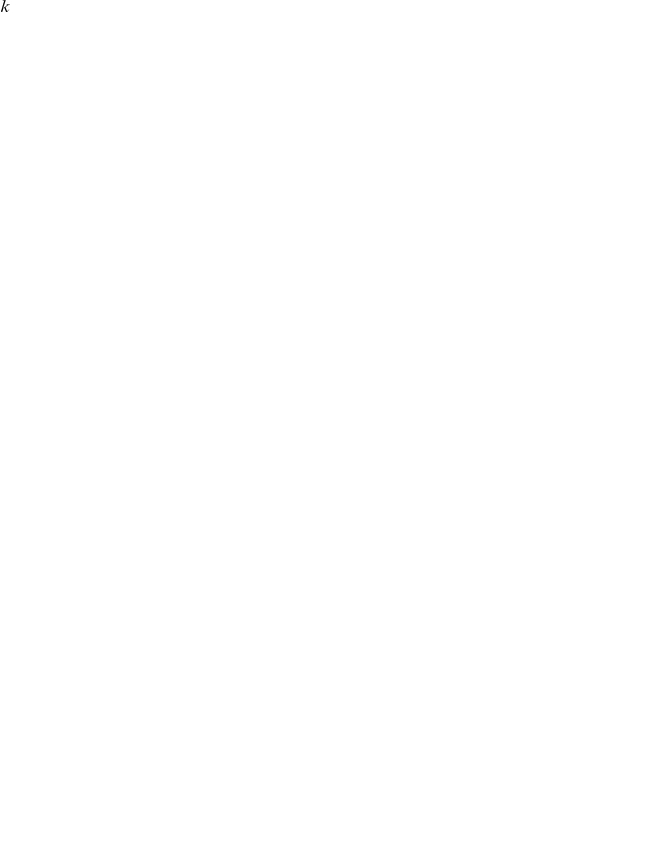
. This means that observed cue-outcome stimuli will have less and less influence onto the associative strength representation, which will quickly converge. Under the dynamic perceptual model however, 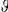 scales the influence the past representation has onto the current one. In other words, it determines the subject's speed of forgetting (discounting): the more volatile the environment, the less weight is assigned to the previous belief (and thus past stimuli) in the current representation. The key difference between the two perceptual models thus reduces to their effective memory.

We (experimentally) estimate the parameter 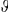 through inversion of the response model 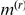, as summarized in the next section. This means the optimisation of perceptual representations has to be repeated for every value of 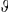 that is considered when observing the observer, i.e. during inversion of the response model. This is an important operational aspect of meta-Bayesian inference, where inversion of the response model entails a nested inversion of the perceptual model.

#### Response model: deciding when to decide

Following the description of the perceptual models, we now define the BDT mapping from representations to behaviour. We assume that subjects decide on the basis of an implicit cost that ranks possible decisions in terms of *what* decision is taken and *when* it is made. This cost is encoded by a loss-function
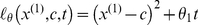
(9)where 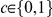 is the subject's choice (*face* or *house*) and 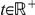 is the decision time. The first term makes a categorisation error costly, whereas the second penalizes decision time. This loss-function creates a speed-accuracy conflict, whose optimal solution depends on the loss parameter 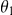. Since the categorization error is binary, the loss parameter 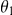 can be understood as the number of errors subjects are willing to trade against one second delay. It is formally an error rate that controls the subject-dependent speed-accuracy trade-off. This can lead to an interaction between observed reaction times and choices, of the sort that explains why people make mistakes when in a hurry (see below).

This loss function is critical for defining optimal decisions: 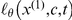 returns the cost incurred by making choice 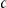 at time 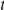 while the outcome category is 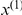. Because subjects experience perceptual uncertainty about the outcome category, the optimal decision 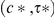 minimizes the *expected loss*, which is also referred to as *posterior risk* 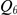 (this is discussed in more detail in the companion paper):
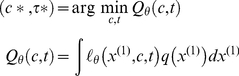
(10)


Note that because the expectation is taken with regard to the posterior density on the hidden states (i.e., the belief about stimulus identity), optimal decisions (concerning both choice 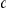 and response time 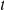) do not only depend on the loss-function 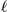, but also on the perceptual model 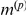.

To derive how posterior risk evolves over time within a trial, we make the representation of outcome category a function of within-trial peristimulus time 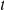 (dropping the trial-specific subscript 
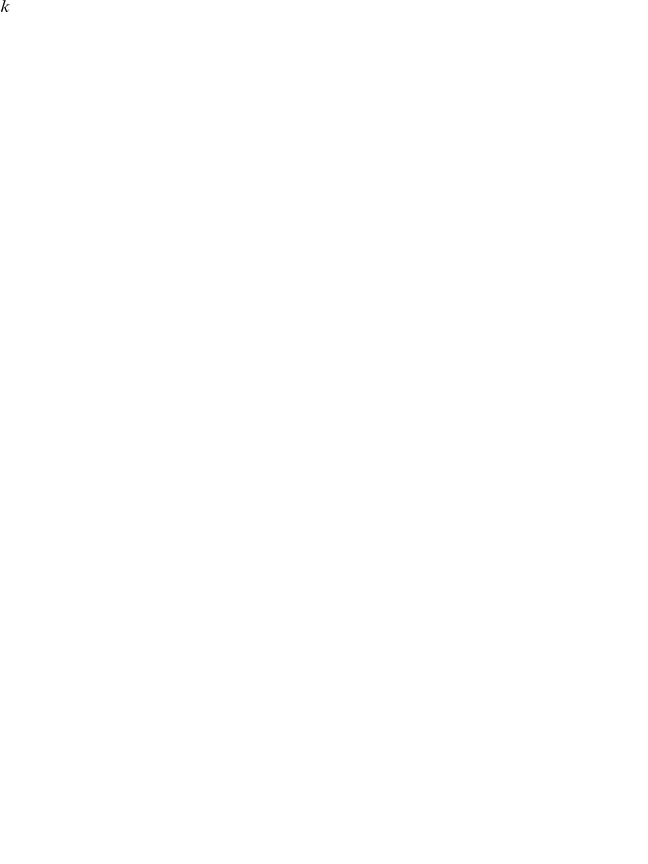
 for clarity): 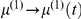. We can motivate the form of 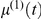 by assuming that the within-trial recognition dynamics derive from a gradient ascent on the perceptual free-energy 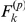. This has been recently suggested as a neurophysiologically plausible implementation of the variational Bayesian approach to perception ([3], [16]; [17]). Put simply, this means that we account for the fact that optimizing the perceptual surprise with respect to the representation takes time.

At each trial, the subject's representation is initialized at her prior prediction 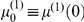, and asymptotically converges to the optimum perceptual free energy 
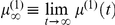
. (Note that the prior prediction at the beginning of a trial, 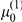, changes over trials due to learning the predictive properties of the auditory cue; see Equations 5–8 above). It turns out (see Appendix S1) that, the posterior risk in Equation 10 can be rewritten as a function of within-trial peristimulus time 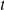 and the difference 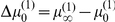 between the posterior representation and the prior prediction of the outcome category (which can thus be thought of as a *post-hoc* prediction error):
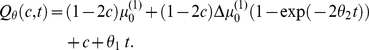
(11)where the second response parameter 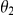 is an unknown scaling factor that controls the sensitivity to *post-hoc* prediction error.

Note that in the present experimental context, the sensory evidence in favour of the outcome category is very strong. Hence, at convergence of the recognition process, there is almost no perceptual uncertainty about the outcome (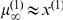). Thus, regardless of the prior prediction 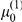, the *post-hoc* prediction error 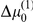 is always positive when a house is presented (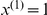) and always negative when a face is shown (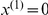). This means that categorization errors occur if: (C1) 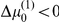 and 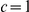, or (C2) 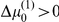 and 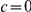. These conditions can be unified by rewriting them as 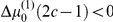 (see the Appendix for further mathematical details). An interesting consequence is that categorization errors can be interpreted as reflecting optimal decision-making: they occur whenever the (learned) prior prediction of the visual outcome is incorrect (e.g. 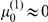 despite 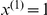) *and* the delay cost is high enough. In other words, categorization errors are optimal decisions if the risk of committing an error quickly is smaller than responding correctly after a longer period.

Note that when 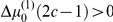 (no categorization error), the posterior risk given in equation 11 is a convex function of decision time 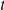. The shape of this convex function is controlled by both the error rate parameter 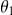 and the sensitivity 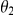 to *post-hoc* prediction error. Finally, Equation 11 yields the optimal reaction time:
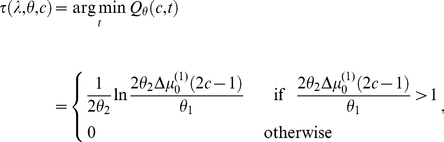
(12)


Note that this equation has two major implications. First, as one would intuit, optimal reaction times and *post-hoc* prediction error show inverse behaviour: as the latter decreases, the former increases. Second, and perhaps less intuitive, the optimal reaction time when committing perceptual categorization errors is zero, because in this case the *post-hoc* prediction error is such that: 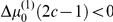. The reader may wonder at this stage whether predicted RTs of zero are at all sensible. It should be noted that this prediction arises from the deterministic nature of Equation 12. When combined with a forward model accounting for random processes like motor noise (see Equation 13 below), non-zero predicted RTs result. Put simply, Equation 12 states that the cost of an error is reduced, if the decision time is very short.

### Inverting the response model

Together with equations 7 and 8, equations 11 and 12 specify the state-space form of our response model 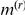:
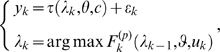
(13)where 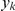 is the observed reaction time at trial 
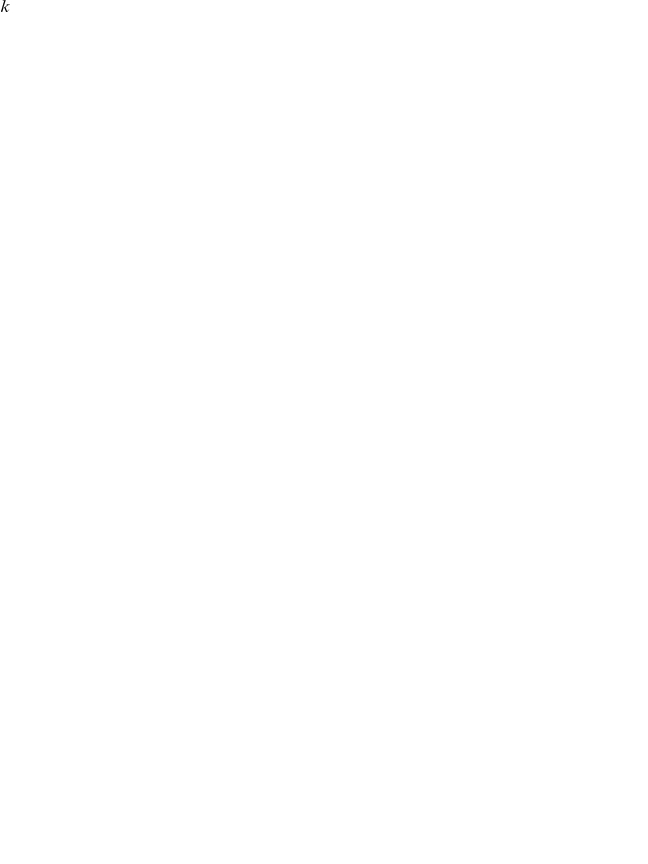
 and the residuals 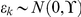, with precision 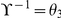, account for (i.i.d. Gaussian) random variability in behavioural responses (e.g. motor noise). The second (evolution) equation models recognition through the minimization of perceptual free energy (or negative sensory surprise) and the first (observation) equation models decision making through the minimization of posterior risk. The functional form of the optimal decision time is given in equation 12 (evaluating the *post-hoc* prediction error 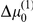 at the current trial) and that of the perceptual free energy is given in equation 5 (recall that learning effects are modulated by the perceptual parameter 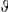). Equation 13 basically implies that the current reaction time 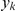 is a nonlinear function of both the response parameters 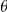 and the perceptual parameter 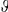, through the history of representations 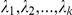. The trial-to-trial variation of reaction times 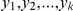 therefore informs us about both the hidden loss and the belief structures of the observer.

The complete formulation of the probabilistic response model involves the definition of the likelihood function (directly derived from equation 13) and the prior density over the unknown model parameters 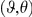. Here, we use weakly informative log-normal priors (see [18]) on the perceptual parameter 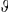 and the response parameters 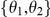 to enforce positivity. These are given in table 1. In addition, the variational Bayesian inversion of the response model makes use of a mean field approximation 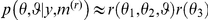 that separates the noise precision parameter 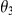 from the remaining parameters. Lastly, we relied on a Laplace approximation to the marginal posterior 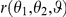. This reduces the Bayesian inversion to finding the first- and second-order moments of the marginal posterior (see equations 13 and 14 in the companion paper [1] for a complete treatment).

**Table 1:** First and second order moments of the prior density over perceptual and response parameters (under both static and dynamical perceptual models).

parameter	prior mean	prior variance
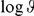 (dynamic perceptual model) (static perceptual model)	02	10^2^10^2^
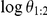	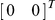	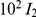
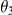	10^4^	10^6^

Note that we used log-normal priors for 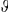 and 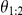, and a Gamma prior for the residuals' precision 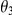.

The algorithmic implementation of the variational Bayesian inversion of the response model is formally identical to that of a Dynamic Causal Model (DCM, see e.g. [19] for a recent review). The variational Bayesian scheme furnishes the approximate marginal posteriors and a lower bound on the response model evidence (via the response free energy 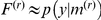), which is used for model comparison. One can also recover the representations since these are a function of the perceptual parameter 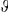, for which we obtain a posterior density 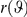 (see equations 12 and 14 in the companion paper [1]):
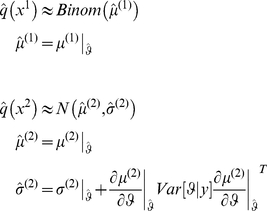
(14)where all sufficient statistics and gradients are evaluated at the mode 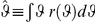 of the approximate posterior 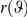 and 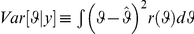 is the experimenter's posterior variance about the perceptual parameter.

## Results

In what follows, we first apply our approach to simulated data in order to establish the face validity of the scheme, both in terms of model comparison and parameter estimation. We then present an analysis of the empirical reaction-time data from the audio-visual associative learning task in [12].

### Monte-Carlo evaluation of model comparison and parameter estimation

We conducted two series of Monte-Carlo simulations (sample size  = 50), under the static (series A) and dynamic perceptual models (series B). In each series, the (log) perceptual parameters were sampled from the intervals 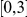 for series A and 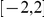 for series B. For both series, the first two (log) response parameters were sampled from the interval 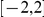. As an additional and orthogonal manipulation, we systematically varied the noise on reaction times across several orders of magnitude: 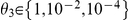. Each simulated experiment comprised a hundred trials and the sequence of stimuli was identical to that used in the real audio-visual associative learning study. We chose the parameters 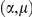 of the perceptual likelihood such that that the discrimination ratio (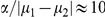) was approximately similar to that of the natural images (see Figure 1). We did not simulate any categorization error. For each synthetic data set, we used both static and dynamic perceptual models for inversion of the response model and evaluated the relative evidence of the perceptual models. Since we knew the ground truth (i.e., which model had generated the data) this allowed us to assess the veracity of model comparison.

**Figure 3:**
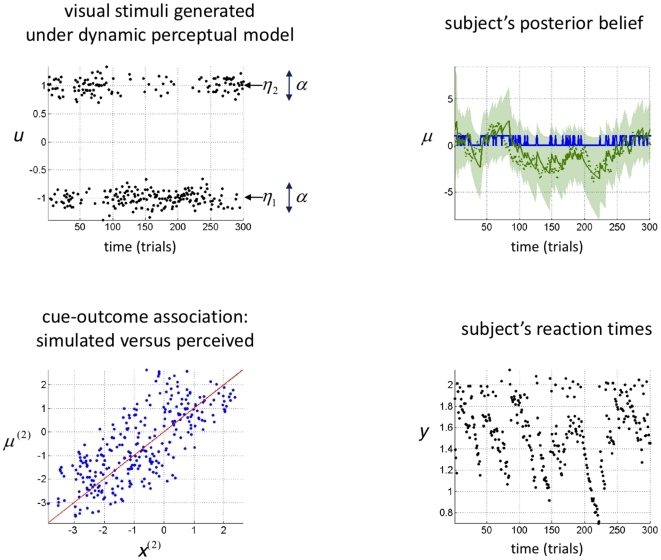
Variational Bayesian recognition of visual stimuli: **Upper Left**: time series of sensory cues, sampled from the generative model summarized in Figure 2. Note that the discrimination ratio (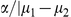) is approximately similar to that of the natural images (see Figure 2). **Upper Right**: Subject's posterior belief, as obtained using the inversion of the perceptual model given observed sensory cues (green: cue-outcome association, blue: visual stimulus category; solid line: posterior mean 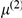, shaded area: 99% posterior confidence interval, dots: sampled hidden states). Note that on each trial, the category of the visual stimuli was recognized perfectly. **Lower Left**: scatter plot comparing the simulated (sampled, x-axis) versus perceived (estimated, y-axis) cue-outcome associative strength. **Lower right**: simulated reaction times.

Figure 3 shows a single example of simulated recognition, in terms of the subject's belief about both the stimulus and the cue-outcome association. For this simulation, the volatility of the association was set to 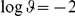 (emulating a subject who assumes a low volatile environment), both for generating stimuli and recognition. We found that the variational Bayesian recognition recovers the stimulus categories perfectly (see blue line in upper-right panel of figure 3) and the cue-outcome association strength well (see lower-left panel and green lines in upper-right panels). This demonstrates that variational recognition is a close approximation to optimal Bayesian inference.

**Figure 4:**
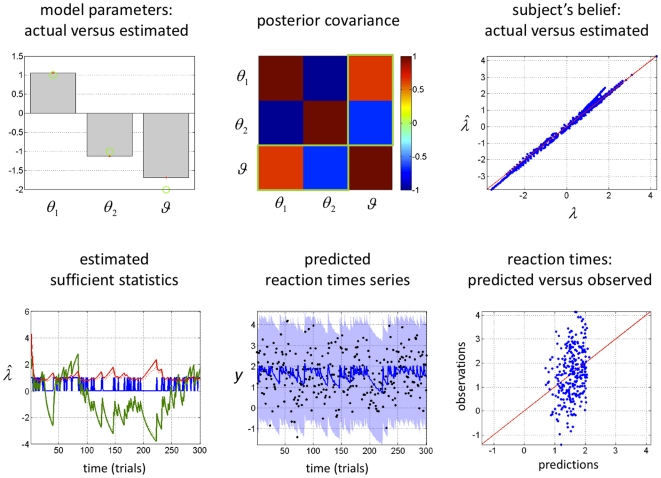
Observing the observer: follow-up example from **Figure 3**. **Upper left**: Comparison between the estimated (grey bars) and actual (green dots) perceptual and response parameters. Note that simulated and estimated parameters are shown in log-space. **Upper centre**: posterior joint correlation matrix of the perceptual and response parameters (the green rectangles depict the correlation between the perceptual parameter 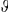 and the response parameters 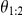). **Upper Right**: scatter plot comparing the simulated (x-axis) and estimated (y-axis) sufficient statistics 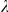 of the approximate subject's posterior. **Lower left**: time series of estimated (solid lines) and simulated (dotted lines) sufficient statistics 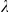 of the approximate subject's posterior (blue: cue identity, green: expected association, red: posterior variance of the associative strength). **Lower centre**: time series of the simulated (black dots) and predicted (solid line: posterior expectation, shaded area: 99% confidence interval) reaction times. **Lower right**: scatter plot comparing the simulated (y-axis) versus predicted (x-axis) reaction times.

Figure 4 shows the inversion of the response model, given the synthetic reaction time data in Figure 3 which were corrupted with unit noise (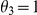). Adding this observation noise yielded a very low signal-to-noise ratio (SNR = 0 dB, see Figure 4), where by definition: 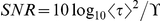. We deliberately used this high noise level because it corresponded roughly to that seen in the empirical data reported below. Table 1 lists the priors we placed on the parameters for this example and for all subsequent inversions with the dynamic perceptual model. Despite the low SNR of the synthetic data, the posterior estimates of the response parameters (grey bars) were very close to the true values (green circles), albeit with a slight overconfidence (upper left panel in Figure 4). Furthermore, the posterior correlation matrix shows that the perceptual and the response parameters are identifiable and separable (upper centre panel). The non-diagonal elements in the posterior covariance matrix measure the degree to which any pair of parameters is non-identifiable (see appendix in the companion paper [1]. Note that the model fit looks rather poor and gives the impression that the RT data are systematically “under-fitted” (lower right and lower centre panels of Fig. 4). This, however, is simply due to the high levels of observation noise: In contrast, the estimation of the true subjective beliefs is precise and accurate (see upper right and lower left panels of Fig. 4). This means that the variational Bayesian model inversion has accurately separated the “observed” reaction time data into noise and signal components. In other words, the estimation of the deterministic trial-by-trial variations of reaction times is not confounded by high levels of observation noise. This result (using simulated data) is important because it lends confidence to subsequent analyses of empirical reaction time data.

**Figure 5:**
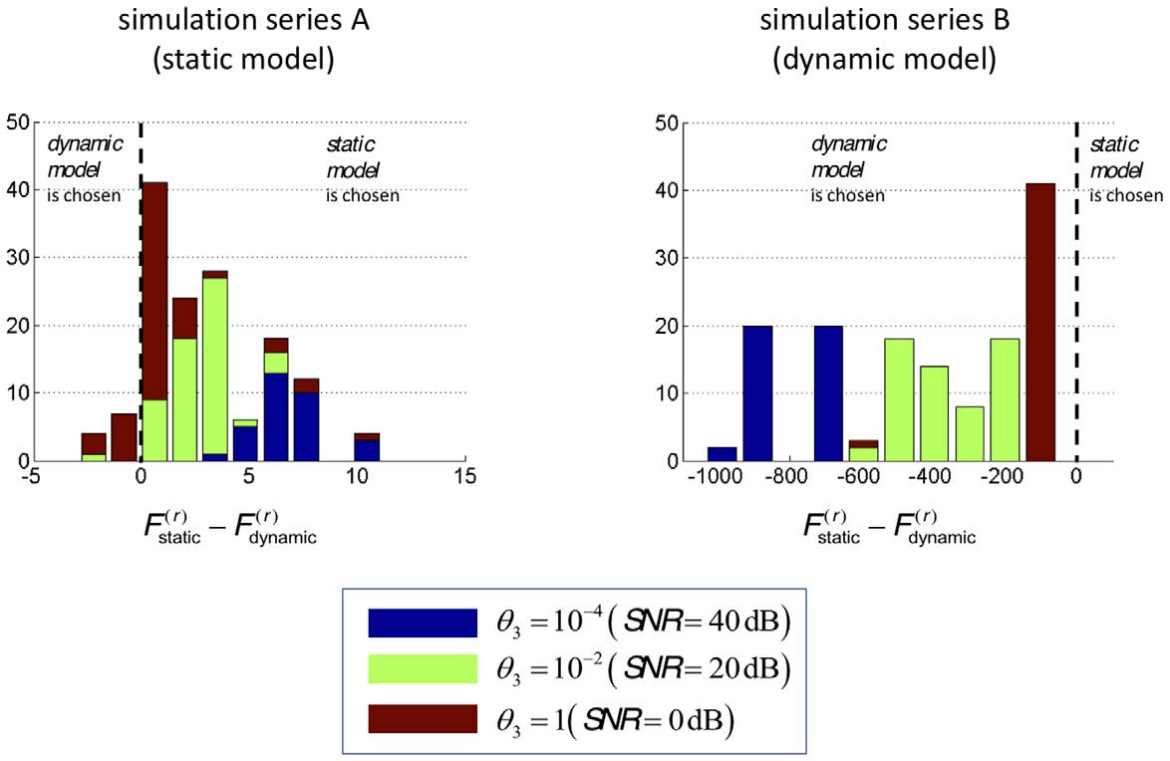
Monte-Carlo empirical distributions of model log-evidence differences (
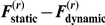
). Blue: SNR  = 40 dB, green: SNR  = 20 dB, red: SNR  = 0 dB. **Left**: Monte-Carlo simulation series A (under the static perceptual model). **Right**: Monte-Carlo simulation series B (under the dynamic perceptual model).

Figure 5 shows the results of the model comparison based on series A and B. This figure shows the Monte-Carlo empirical distribution of the response free-energy differences 
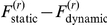
, where 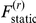 (respectively 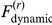) is the approximate log-evidence for the response model under the static (respectively dynamic) perceptual model. This relative log-evidence is the approximate log-Bayes factor or log odds ratio of the two models. It can be seen from the graphs in Figure 5 that model comparison identifies the correct perceptual model with only few exceptions for the static model (left panel) and always for the dynamic model (note that a log-evidence difference of zero corresponds to identical evidence for both models). Table 2 provides the average free-energy differences over simulations as a function of the true model (simulation series) and SNR.

**Table 2:** Monte-Carlo averages of log-evidence differences as a function of simulation series (A: static and B: dynamic) and SNR.

	Series A (static) 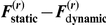	Series B (dynamic) 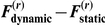
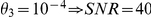 dB	7.52	752.9
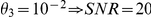 dB	3.22	320.7
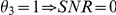 dB	1.86	28.6

It is interesting that the free-energy differences are two orders of magnitude larger for series B, relative to series A. In other words, when the data-generating model is the dynamic one, it is easier to identify the true model from reaction times than when the static model generated the data. This might be due to the fact that the static model is a limiting case of the dynamic model; i.e. when the volatility 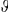 tends to zero the dynamical perceptual model can account for the variability in reaction times generated using the static model. However, note that this difference in model complexity does not distort or bias our model comparisons since the free energy approximation to the model evidence accounts for such differences in complexity [20].

As expected there is also a clear effect of noise: the higher the SNR, the larger the relative log-evidences. This means that model comparison will disambiguate models more easily the more precise the experimental data.

**Figure 6:**
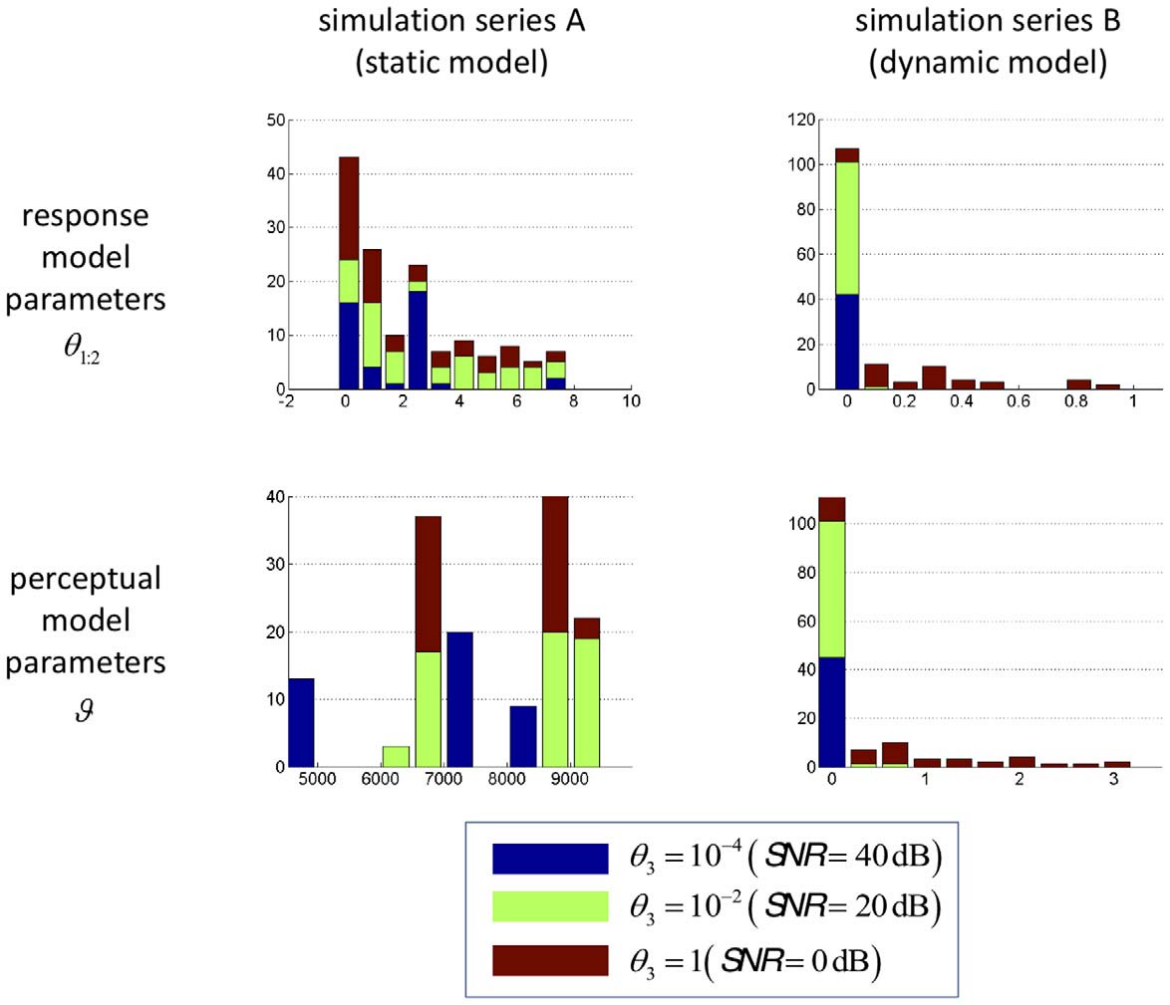
Monte-Carlo empirical distributions of the parameter estimation error (SSE score). Blue: SNR  = 40 dB, green: SNR  = 20 dB, red: SNR  = 0 dB. **Upper left**: response model parameters, Monte-Carlo simulation series A. **Upper right**: response model parameters, Monte-Carlo simulation series B. **Lower left**: perceptual model parameters, Monte-Carlo simulation series A. **Lower right**: perceptual model parameters, Monte-Carlo simulation series B. Note that the SSE score was evaluated in log-space.

We next characterised the accuracy of parameter estimation under the best (correct) model, using the sum of squared error (SSE), in relation to the true values. We computed the Monte-Carlo empirical distribution of the SSE for each set of (perceptual and response) parameters, for each simulation series (A and B) and SNR. Figure 6 shows these distributions and Table 3 provides the Monte-Carlo averages.

**Table 3:** Monte-Carlo averages of the SSE as a function of simulation series (A or B) and SNR, for perceptual and response parameters.

		Series A (Static)	Series B (Dynamic)
perceptual parameters	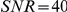 dB	7.8 10^3^	6.0 10^−4^
	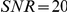 dB	8.4 10^3^	6.1 10^−2^
	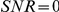 dB	8.2 10^3^	1.09
response parameters	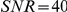 dB	2.20	2.0 10^−4^
	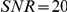 dB	3.39	2.1 10^−2^
	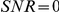 dB	2.38	3.5 10^−1^

Quantitatively, the parameters are estimated reasonably accurately, except for the perceptual parameter (prior precision on associative strength) of the static model. This reflects the fact that the prior on association strength has little impact on the long-term behaviour of beliefs, and hence on reaction times. This is because within the static perceptual model, 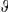 acts as an initial condition for the dynamics of the representation 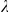, which are driven by a fixed point attractor that is asymptotically independent of 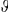. Thus, only the first trials are sensitive to 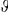. The ensuing weak identifiability of 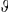 expressed itself as a high estimation error (high SSE). Again, there is a clear effect of noise, such that the estimation becomes more accurate when SNR increases. Also, consistent with the model comparison results above, parameter estimates are more precise for the dynamic model than for the static one.

### Application to empirical reaction times

The Monte-Carlo simulations above demonstrate the face validity of the method, in the sense that one obtains veridical model comparisons and parameter estimates, given reaction time data with realistic SNR. We now apply the same analysis to empirical reaction times from nineteen subjects [12]. Specifically, we hoped to show two things to provide evidence for the construct validity of our approach: first, that the dynamic model (which was consistent with the information given to the subjects) would have higher evidence than the static model (which was not), and secondly, that our results would generalise over both auditory cues, both in terms of model comparison and parameter estimates (as explained above, we treated reaction times for the two cues as separate sequences).

We conducted a hierarchical (two-level) analysis of the data from the nineteen subjects. Note that the original study by [12] contained twenty subjects. For experimental reasons, one of these subjects experienced a different stimulus sequence than the rest of the group. Even though it would have been perfectly possible to analyze this subject with the present approach, we decided, for reasons of homogeneity in the inter-subject comparison, to focus on subjects with identical stimulus sequence. In a first-level analysis, we inverted both dynamic and static models on both type I cues (high pitch tones) and type II cues (low pitched tones) separately, for each subject. As in the simulations above, the parameters 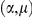 of the perceptual likelihood (equation 1) were chosen such that stimulus discriminability (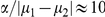) was similar to that of the natural images (see Figure 1). Also, categorization errors were assigned a response time of zero (see histograms in upper right panels of Figs. 12–13) and a very low precision 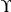, relative to the other trials. This allowed us to effectively remove these trials from the data without affecting the trial-to-trial learning effects.

**Figure 7:**
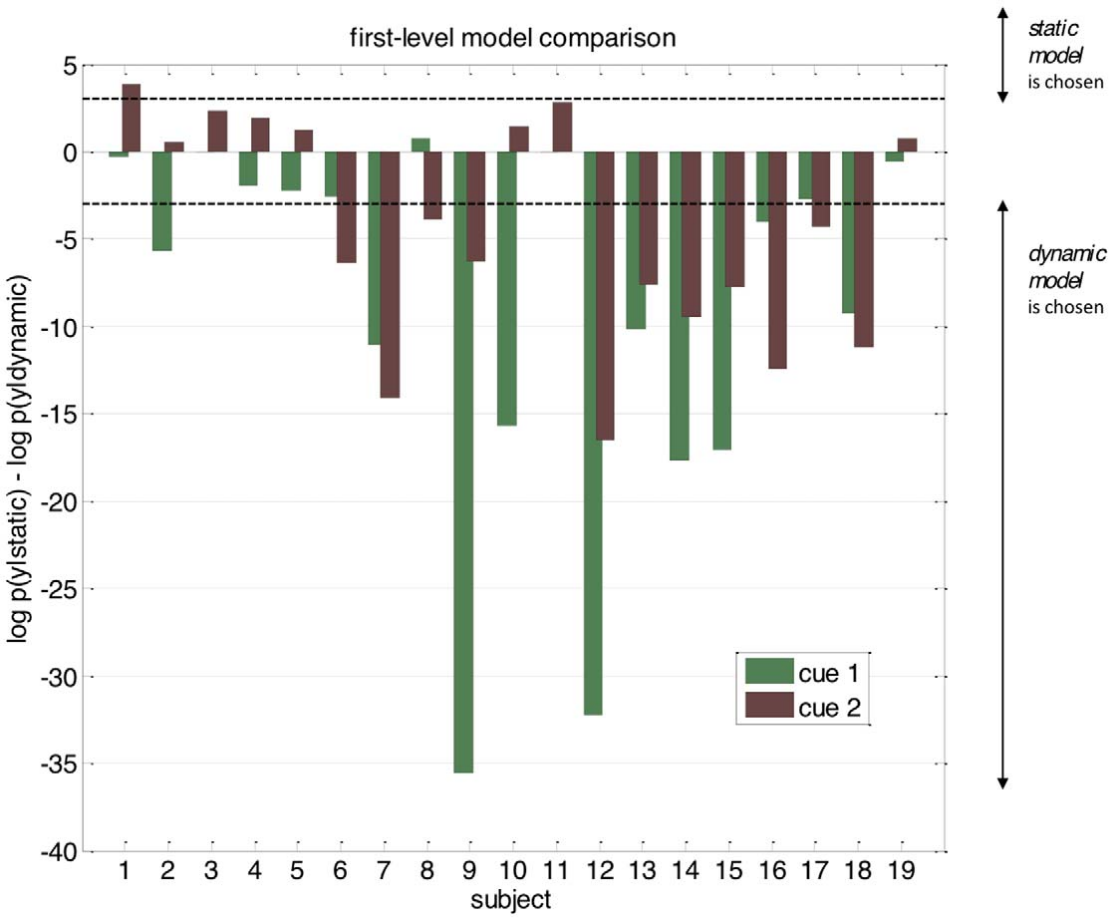
Subject-level model comparison. The graph is a bar plot of the difference in model evidence for the static model versus the dynamic model (
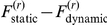
), for each subject (along the x-axis) and each cue (green: type I cue, red: type II cue).

Figure 7 summarizes the model comparison results for each subject, showing the difference in log-evidence for both auditory cues. A log-evidence difference of three (and higher) is commonly considered as providing strong evidence for the superiority of one model over another [21]. Using this conventional threshold, we found that in 13 subjects out of 19 the competing perceptual models could be disambiguated clearly for at least one cue type. It can be seen that for all of these subjects except one the dynamic perceptual model was favoured. Also, it was reassuring to find that the variability of response model evidences across cue types was much lower than its variability across subjects. In particular, in 10 out of the 13 subjects where the perceptual models could be distinguished clearly, the model evidences were consistent across cue types.

**Figure 8:**
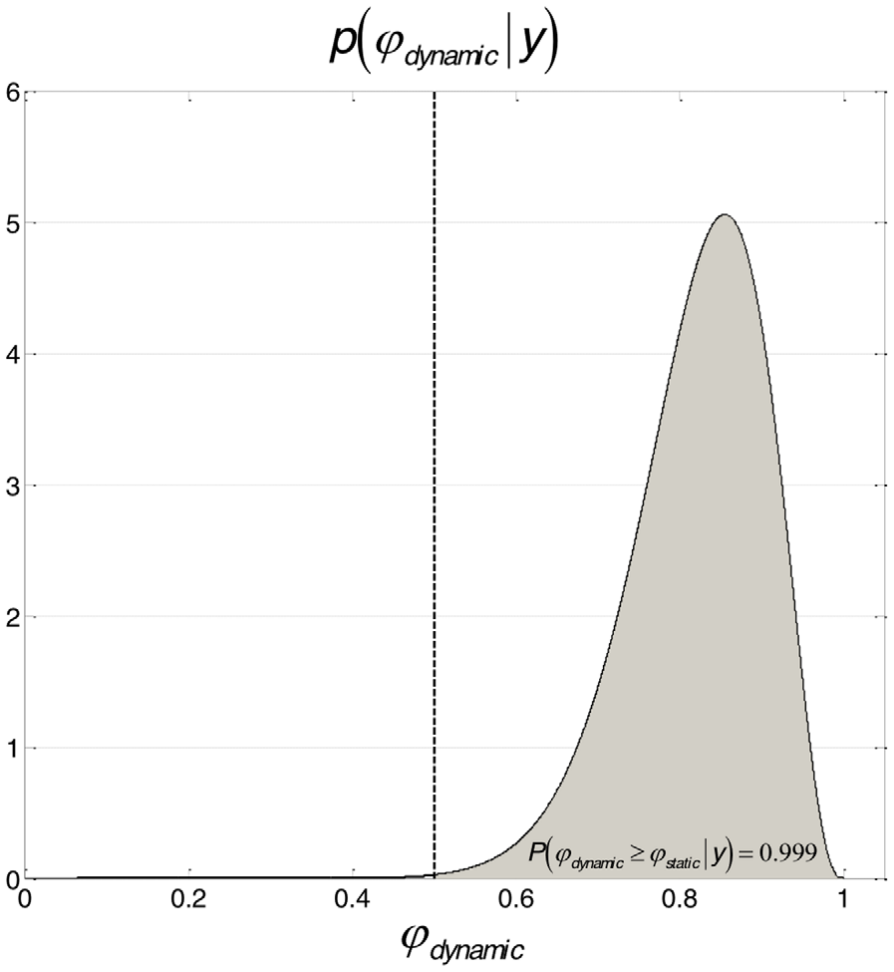
Group-level model comparison. Dirichlet posterior distribution of the frequency (within the group of subjects) 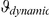 of the dynamic model, given all subjects data 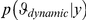. The grey area depicts the exceedance probability 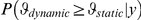, i.e. the probability that the dynamic model is more likely (within the group) that the static one.

In a second step, we performed a Bayesian group-level random effect analysis of model evidences [20]. Assuming that each subject might have randomly chosen any of the two perceptual models, but consistently so for both cues, we used the sum of the subject-specific log-evidences over both cues for model comparison at the group level. Figure 8 shows the ensuing posterior Dirichlet distribution of the probability 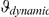 of the dynamic perceptual model across the group, given all datasets. Its posterior expectation was approximately 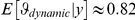. This indicates how frequently the dynamic model won the model comparison within the group, taking into account how discernable these models were. We also report the so-called “exceedance probability” of the dynamic model being more likely than the static model, given all datasets: 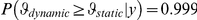. This measures the overall strength of evidence in favour of the dynamic perceptual model, at the group level. This is a pleasing result because, as described above, the dynamic model (where subjects assume *a priori* that the cue-outcome association is varying in time) was consistent with the information delivered to the subjects (whereas the static model was not).

**Figure 9:**
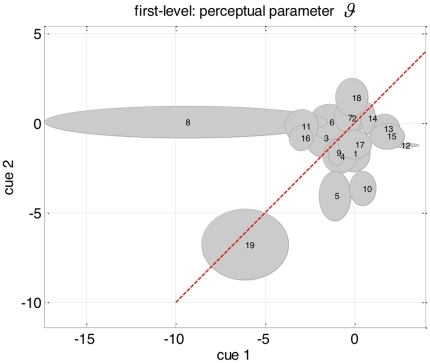
Plot of the reliability of perceptual parameter estimates 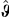 across cue types. The perceptual parameter estimate 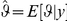 and its posterior variance 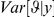 are plotted as a function of cue types (on the x and y axis) and shown as an ellipse for each subject. The centre of the ellipse represents 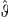 for each cue, and its vertical and horizontal axis show one posterior standard deviation around it. The red line shows the ideal positions of the parameter estimates (the centre of the ellipses) if there was perfect reliability (i.e. no variability across cue types).

**Figure 10:**
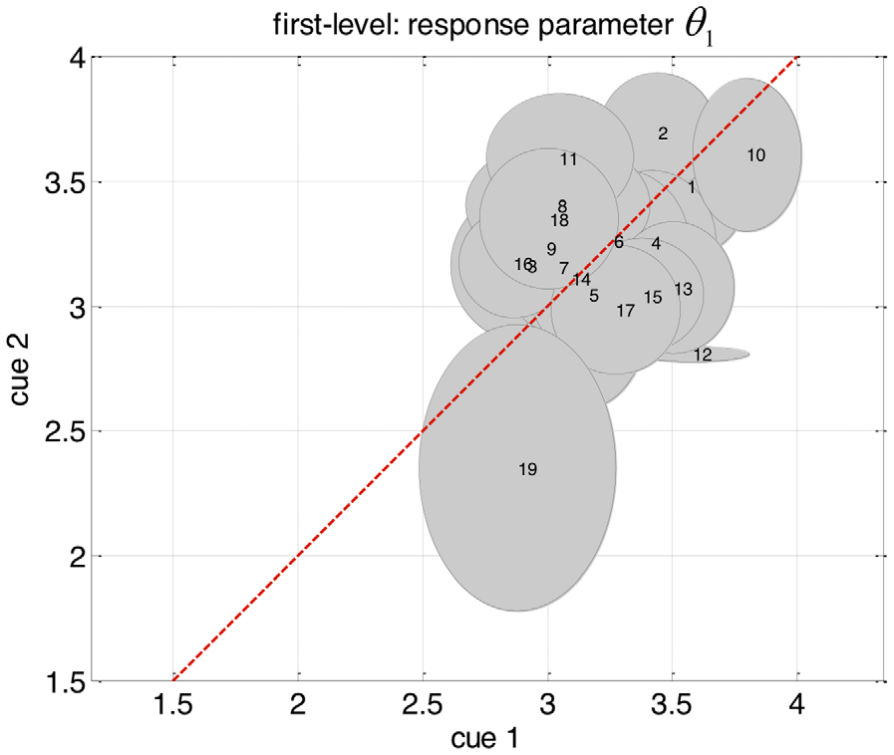
Plot of the reliability of response parameter estimates 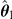. See legend to Fig. 9 for explanations.

**Figure 11:**
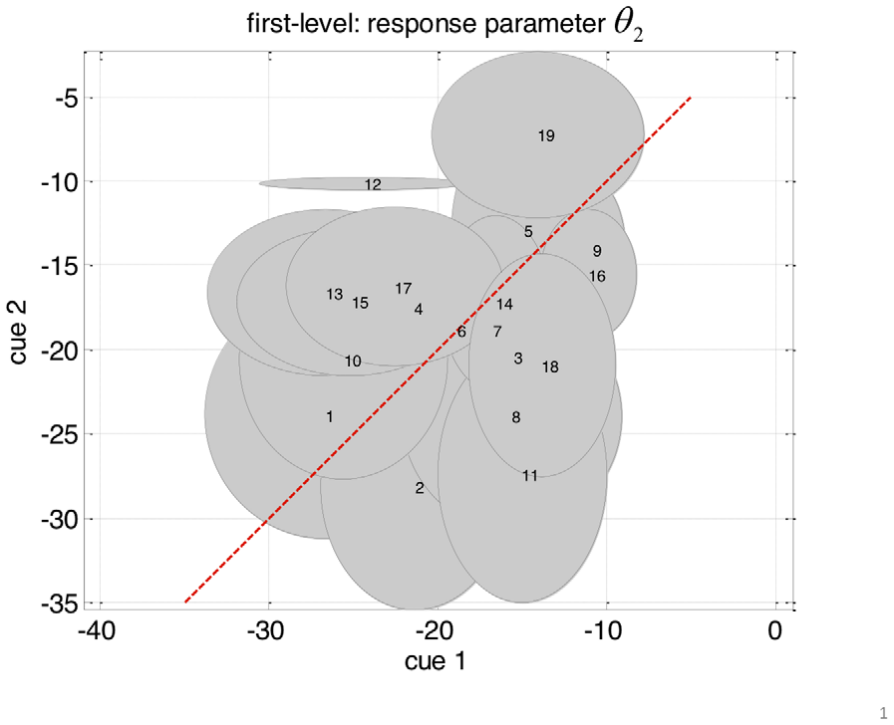
Plot of the reliability of response parameter estimates 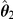. See legend to Fig. 9 for explanations.

Having established the dynamic model as the more likely model of reaction time data at the group level, we now focus on the actual estimates of both response and perceptual parameters. First, we tested for the reliability of the parameter estimates, that is, we asked whether the subject-dependent posterior densities 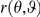 of the perceptual and response parameters were reproducible across both types of cues. Specifically, we hoped to see that the variability across both types of cues was smaller than the variability across subjects. For the three parameters 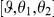
_,_ figures 9, 10 and 11 display the variability of the posterior densities across both cues and all subjects, taking into account the posterior uncertainty 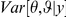 (see equation 14). First, it can be seen that there is a consistent relationship between cue-dependent parameter estimates. Second, there is a comparatively higher dispersion of parameter estimates across subjects than across cues. Taken together, this demonstrates the reliability of parameter estimates in the context of empirically measured behavioural data with low SNR (i,e., reaction times). This implies that one can obtain robust and subject-specific inferences with our approach.

**Figure 12:**
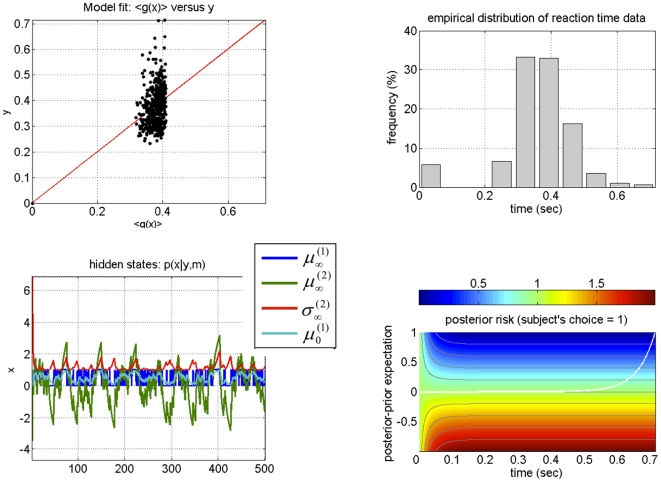
Results of inverting the response model for subject 5. Upper-left: predicted (x-axis) versus observed (y-axis) reaction time data. The red line indicates perfect agreement between the model and the data. Upper-right: observed reaction times empirical histogram. Note that incorrect decisions were assigned a response time of zero and did not influence model fit; see main text for details. Lower-left: time series (trial-by-trial) of the sufficient statistics of subject 5's representations of both the outcome category (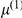) and associative strength (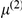 and 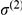). See main text for the precise meaning of these variables. Lower-right: posterior risk as a function of *post-hoc* prediction error (y-axis), i.e. the difference between posterior and prior expectations, and decision time (x-axis). The posterior risk is evaluated at subject 5's response parameters estimate 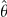 for ‘house’ decisisions (i.e. 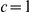); it can be symmetrically derived for 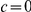. The white line shows the optimal decision time 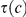 for each level of *post-hoc* prediction error (see Equation 12 in the main text). Note that 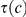 is identically zero for all negative *post-hoc* prediction error. This signals a perceptual categorization error (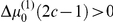, see Equation 11 in main text), which is emitted (at the limit) instantaneously.

**Figure 13:**
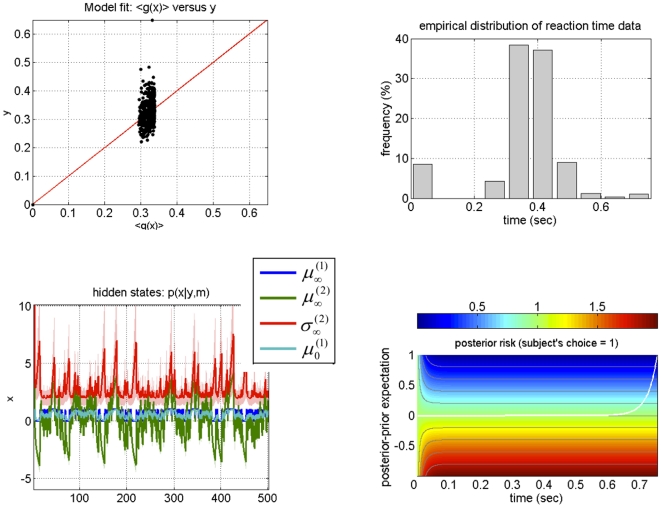
Results of inverting the response model for subject 12. See legend to Fig. 12 for details.

Such inferences concern both subject-specific a priori beliefs (e.g., about the stability of the environment; see equations 3 and 4) and preferences (as encoded by their individual loss function; see equation 9). To demonstrate the potential of our approach for characterizing inter-individual differences in beliefs and preferences, we present a subject-specific summary of the inverted response model (under the dynamic perceptual model) for two individuals (subjects 5 and 12). These results are summarized by Figures 12 and 13.

First, we would like to stress that, as for the group as a whole, the SNR of empirical data from these two subjects is similar to the SNR of the Monte Carlo simulation series described above (around 0 dB; see Figure 4). It is therefore not surprising that the model fit to the empirical looks similarly bad as in our simulations (compare upper left panels in Figs. 12-13 with lower right panel in Fig. 4). Note, however, that our simulations demonstrated that despite this poor fit the model parameters were estimated with high accuracy and precision; this instils confidence in the analysis of the empirical data.

Even though the two histograms of reaction time data from these two subjects were almost identical (compare upper right panels in figures 12 and 13), the trial-to-trial variations of reaction time data allowed us to identify rather different subject-specific structures of beliefs and preferences (loss functions). Concerning the beliefs of these two individuals, the parameter estimates indicated that, a priori, subject 5 assumed a much more stable environment (i.e., had a much lower prior volatility 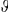) than subject 12; as a consequence, the dynamics of her estimates of the associative strength 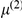 are considerably smoother across trials (compare lower left panels in figures 12 and 13). In other words, she averaged over more past cue-outcome samples when updating her posterior belief or representation than subject 12. Another consequence of this is the fact that subject 5 uncertainty 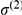 about the associative strength is much smaller and less “spiky” than subject 12's. This has an intuitive interpretation: since subject 12 assumes a volatile environment, a series of predicted visual outcomes (approaching a nearly deterministic association) is highly surprising to her. This causes high perceptual uncertainty about the tracked associative strength whenever its trial-to-trial difference approaches zero.

As for the preferences (loss functions) that guided the actions of these two subjects, subject 12 displays a greater variability in her optimal decision times for very small post-hoc prediction errors (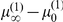, see equation 12). As a consequence, her optimal decision time is greater than that of subject 5, for any given magnitude of the *post-hoc* prediction error (compare lower right panels in Figures 12 and 13). This is because both subject 12's error rate (i.e., 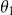) and sensitivity to *post-hoc* prediction error (i.e., 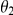) is smaller than subject 5's.

In summary, subject 12 is assuming a more variable associative strength. This means that, when compared to subject 5, she discards information about past cue-outcome associations more quickly and has more uncertain (prior) predictions about the next outcome. However, she is willing to make more categorization errors per second delay than subject 5. This is important, since she effectively needs more time to update her uncertain (i.e. potentially inaccurate) prior prediction to arrive at a correct representation. In contrast, subject 5 is more confident about her prior predictions and is more willing to risk categorization errors in order to gain time.

## Discussion

In a companion paper [1], we have described a variational Bayesian framework for approximating the solution to the Inverse Bayesian Decision Theory (IBDT) problem in the context of perception, learning and decision-making studies. We propose a generic statistical framework for (i) comparing different combinations of perceptual and response models and (ii) estimating the posterior distributions of their parameters. Effectively, our approach represents a meta-Bayesian procedure which allows for Bayesian inferences about subject's Bayesian inferences. In this paper, we have demonstrated this approach by applying it to a simple perceptual categorization task that drew on audio-visual associative learning. We have focused on the problem of ‘deciding when to decide’, i.e. we have modelled reaction time data as arising from subjective beliefs and preferences under the constraint of a speed-accuracy trade-off. This model is novel and quite different from classical evidence accumulation and ‘race’ models (e.g. [22], [23], [10]), in two ways. First, a reaction time is understood in terms of the convergence speed of an optimization process, i.e. perceptual recognition. This is because it takes time for a (variational) Bayesian observer to arrive at an optimal representation or belief. In this paper, the within-trial (peri-stimulus time) dynamics of the recognition process emerged from a gradient-ascent on the free-energy, where free-energy is a proxy for (negative) perceptual surprise under a given perceptual model. The resulting form of the response model is analytically tractable and easy to interpret. Second, the variability of reaction times across subjects is assumed to depend on individual differences in prior beliefs (e.g., about the stability of the environment) and preferences (i.e., loss or utility functions). Our approach thus provides insights into both within-trial mechanisms of perception as about inter-individual differences in beliefs and preferences.

In this work, we have chosen to focus on modelling reaction time data and have deliberately ignored categorization errors. This is because considering both reaction time and choice data at the same time would have required an extension of the response likelihood. The difficulty here is purely technical: the ensuing bivariate distribution is Bernoulli-Gaussian, whose sufficient statistics follow from the posterior risk (equation 11). Although feasible, deriving this extended response model would have significantly increased its complexity. Since the focus of this article was to provide a straightforward demonstration of our theoretical framework (described in the companion paper), we decided not to include choice data in the response model. Clearly, this is a limitation as we are not fully exploiting the potential information about underlying beliefs and preferences that is provided by observed categorization errors. This extension will be considered in future work.

In our model, categorization errors arise when incorrect prior predictions coincide with high delay costs (see equations 11 and 12). One might think that there is an irreconcilable difference between this deterministic scheme and stochastic diffusion models of binary decisions ([24]; see also [25], for a related Bayesian treatment). However, there are several ways in which our scheme and stochastic diffusion models can be reconciled. For example, the trial-wise deterministic nature of our scheme can be obtained by choosing the initial condition of the stochastic process such that the probability of reaching the upper or lower decision threshold is systematically biased in a trial-by-trial fashion. Also, delay costs can be modelled by letting the distance between lower and upper diffusion bounds shrink over time. Alternatively, one could motivate the form of stochastic diffusion models by assuming that the brain performs a stochastic (ensemble) gradient ascent on the free energy. This would relate the frequency of categorization errors to task difficulty; for example, when a stimulus is highly ambiguous or uncertain, the perceptual free energy landscape is flat (perceptual uncertainty is related to the local curvature of perceptual free energy; see equations 5 and 6 of the companion paper). In summary, there are several ways in which our approach and stochastic diffusion models could be formally related. The utility of such hybrid models for explaining speed-accuracy trade-offs (cf. [26]) will be explored in future work.

We initially evaluated the method using Monte-Carlo simulations under different noise levels, focusing on model inversion given synthetic data and on how well alternative models could be disambiguated. This enabled us to assess both the efficiency of parameter estimation and veracity of model comparison as a function of SNR. Importantly, we found that even under very high noise levels (SNR = 0dB, comparable to the SNR of our empirical data), and therefore poor model fit, the model nevertheless (i) yielded efficient estimates of parameters, enabling us to infer and track the trial-to-trial dynamics of subjective beliefs from reaction time data, and (ii) robustly disambiguated correct and wrong models. We then applied the approach to empirical reaction times from 19 subjects performing an associative learning task, demonstrating that both model selection results and parameter estimates could be replicated across different cue types. Reassuringly, the model selection results were consistent with the information available to the subjects. In addition, we have shown that subject-to-subject variability in reaction times can be captured by significant differences in parameter estimates (consistently again across cue types) where these parameters encode the prior beliefs and preferences (loss functions) of subjects,

Together, the simulations and empirical analyses establish the construct validity of our approach and illustrate the type of inference that can be made about subjects' priors and loss-functions. Our results suggest that the approach may be fairly efficient when it comes to comparing and identifying models of learning and decision-making on the basis of (noisy) behavioural data such as reaction times.

Some readers may wonder why we have used a relatively complicated criterion to evaluate the relative goodness of competing models; i.e., an approximation to the log-evidence, instead of simply comparing their relative fit. Generally, pure model fit indices are not appropriate for comparing models and should be avoided (cf. [27]-[29]). There are many reasons why a perfectly reasonable model may fit a particular data set poorly; for example, independent observation noise (see Figure 4 for an example). On the other hand, it is easy to construct complex models with excellent or even perfect fit, which are mechanistically meaningless and do not generalize (i.e., “over-fitting”). In brief, competing models cannot be compared on the basis of their fit alone; instead, their relative complexity must also be taken into account. This is exactly what is furnished by the (log) model evidence, which reports the balance between model fit and complexity (and can be approximated efficiently by the variational techniques used in this paper). This allows us to compare models of different complexity in an unbiased fashion. Crucially, our Bayesian model selection method does not require models to be nested and does not impose any other constraints on the sorts of model that can be compared ([30], [20]). For example, alternative models compared within our framework could differ with regard to the mathematical form of the perceptual or the response model, the priors or the loss function – or any combination thereof. In principle, this makes it possible to investigate the relative plausibility of different explanations: For example, whether individual differences in behaviour are more likely to result from individual differences in the perceptual or the response model. For clarity, however, the empirical example shown in this paper dealt with a very simple case, in which the perceptual model was varied while the response model was kept fixed.

As with all inverse problems, the identifiability of the BDT model parameters depends upon both the form of the model and the experimental design. In our example, we estimated only one parameter of the perceptual models we considered. One might argue that rather than fixing the sensory precision (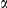, see Equation 1) with infinitely precise priors, we should have tried to estimate it from the reaction time data. It turns out, however, that estimating 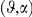 and 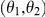 together represents a badly conditioned problem; i.e. the parameters are not jointly identifiable because of posterior correlations among the estimates. This speaks to the utility of generative models for decision-making: the impact that their form and parameterisation has on posterior correlations can be identified before any data are acquired. Put simply, if two parameters affect the prediction of data in a similar way, their unique estimation will be less efficient. In our particular example, there is no critical need to estimate 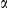 from the data. This is because faces and houses are well-known objects for whose categorisation subjects have a life-long experience. It is therefore reasonable to assume that 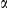 is known to the subjects, and its value can be chosen in correspondence with the statistics of the visual stimuli (see above). However, pronounced inter-individual differences can be observed empirically in face-house discrimination tasks, and this may result from differences in the individuals' history of exposure to faces throughout life. A limitation of our model is that it does not account for such inter-subject variability but assumes that 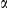 is fixed across subjects.

In contrast to 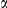, which can (and should) be treated as a fixed parameter, it is necessary to estimate the perceptual parameter 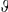. Note that from the subject's perspective, 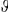 (similar to 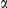) is quasi-fixed (i.e., with nearly infinite precision) as this prior has been learnt throughout life. From the experimenter's perspective, however, 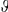 is an unknown parameter which has to be inferred from the subject's behaviour. Estimating this parameter is critical for the experimenter as its value determines the subject's learning rate. This is best explained by highlighting the link between ‘learning rates’, as employed by reinforcement learning models, and Bayesian priors, or more precisely *prior precision parameters*. In the ‘dynamic’ perceptual model, the learning rule effectively replaces the past history of sensory signals with a summary based on the previous representation (see Eq. 8). In turn, the perceptual representation discounts past sensory signals with an exponential weighting function, whose half-life is an affine transformation of the prior volatility 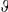. The link between 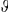 and the subject's learning rate can be seen by considering the solution to equation 8 (at convergence):
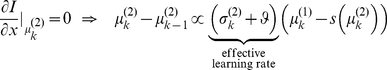
(15)where 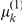 is the belief about the auditory outcome category (face/house) and 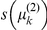 is its posterior prediction based on the past history of sensory signals. Equation 15 gives the effective update rule for the perceived associative strength 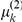 when the perceptual free energy has been optimized. Note that the form of Eq. 15 corresponds to the Rescorla-Wagner learning rule [31], in which the change in associative strength 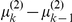 is proportional to the prediction error, i.e. 
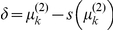
.

In summary, for the model in the present paper, the subject's learning rate depends on the prior volatility 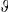 of cue-outcome associations. Note, however, that there may not always be a quantitative relation between prior precision parameters and learning rates because this depends on the specificities of the perceptual model. There is a general qualitative relationship between the two quantities, however, because the prior precision of hidden causes within hierarchical perceptual models controls the relative weight of upcoming sensory information and prior (past) beliefs in forming the actual posterior representation. In short, this means the learning rate itself (and thus any ‘forgetting’ effect) emerges from optimal Bayesian recognition (see e.g., [32] for a nice example). A full treatment of these issues will be presented in forthcoming work [33].

Another analogy concerns the optimal decision time derived from the speed-accuracy trade-off given in Equation 12 which is similar in form to Hick's law. This law relates the reaction times to the amount of extracted information (c.f. [34]). In its simplest form, Hick's law is given by: 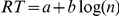, where 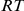 is the expected reaction time and 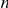 is the number of choice alternatives. Here, 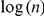 is the perceptual uncertainty (as measured by Shannon entropy). It turns out that when no categorization error is made, Equation 12 could be rewritten as 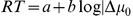, where 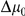 is the *post-hoc* prediction error, i.e. posterior minus prior expectation. Put simply, 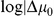 measures incoming information. There are obvious formal (information theoretic) differences between Equation 12 and Hick's law, but they capture similar intuitions about the mechanisms causing variations in reaction times.

This paper has demonstrated the practical application of the meta-Bayesian framework described in the companion paper, using empirical reaction time data from an audio-visual associative learning task reported in [12]. Authors presented several analyses of these data, including a formal comparison of alternative learning models. The results provided in the present article finesse the original comparisons and take us substantially beyond the previous report. First, the paper [12] did not provide any decision theoretic explanation for (learning induced) motor facilitation. In that paper, the behavioural comparison of different learning models was a precursor to using prediction error estimates in a model of fMRI data. It therefore only used a very simple response model assuming that (inverse) reaction times scale linearly with prediction error. In contrast, we have proposed a response model that is fully grounded in decision theory and does not assume a specific (e.g., logarithmic) relationship between prediction errors and motor facilitation. Second, we conducted a full two-level analysis of the reaction time data, in order to assess inter-individual differences. This was made possible because, as opposed to the work in [12], we allowed for inter-individual differences in both the perceptual and response parameters (see above).

Finally, we wish to emphasize that the “observing the observer” (OTO) approach for inference on hidden states and parameters can be obtained in a subject-specific fashion, as demonstrated by our empirical analyses in this paper (see Figs. 9-13). This allows for analyses of inter-individual differences in the mechanisms that generate observed behaviour. Such quantitative inference on subject-specific mechanisms is not only crucial for characterizing inter-individual differences, an important theme in psychology and economics in general, but also holds promise for clinical applications. This is because spectrum diseases in psychiatry, such as schizophrenia or depression, display profound heterogeneity with regard to the underlying pathophysiological mechanisms, requiring the development of models that can infer subject-specific mechanisms from neurophysiological and/or behavioural data [35]. In this context, the approach presented in this paper can be seen as a complement to DCM: OTO may be useful for inference on subject-specific mechanisms expressed through behaviour, in a similar way as DCM is being used for inference on subject-specific mechanisms underlying neurophysiology.

## Supporting Information

Appendix S1 Appendix S1 (‘*deciding when to decide*’) is included as ‘supplementary material’. It summarizes the mathematical derivation of the optimal reaction times (as given in equation 12) from first principles, within the framework of Bayesian Decision Theory.
(DOC)
