# Supplementary material for: Observing the Observer (II): Deciding When to Decide
Source: PLoS One. 2010 Dec 14;5(12):e15555. doi: 10.1371/journal.pone.0015555 (PMC3001882; doi:10.1371/journal.pone.0015555)
Supplement: Appendix S1 — (‘deciding when to decide’) is included as ‘supplementary material’. It summarizes the mathematical derivation of the optimal reaction times (as given in equation 12) from first principles, within the framework of Bayesian Decision Theory. (DOC) [file pone.0015555.s001.doc]

**Appendix S1: deciding when to decide**

As detailed in the main text, the paradigm we deal with is a combination of a perceptual categorization task, where subjects are presented with a visual stimulus (house or face) and are asked to classify it as quickly as possible, and an associative learning task, where each of the visual stimuli is preceded by an auditory cue signalling the nature of the visual stimulus with a certain probability. Critically, the predictive strength of the auditory cue varies over time. By learning the current audio-visual cue-outcome association strength and its variability over time (i.e., volatility of the environment), subjects can predict the visual stimulus and thus speed up its classification.

We have dropped the trial index in the remainder of the Appendix for the sake of simplicity. On each trial, the sensory signal consists of an image (denoted by $u$), which can belong to two classes (denoted by $x^{(1)}$, where $x^{(1)}=0$ means a face and $x^{(1)}=1$ means a house). The subject’s prior belief about $x^{(1)}$ comes from a prediction derived from the learned cue-outcome association (see above and main text), and starts to evolve into a posterior belief as soon as $u$ is observed.

The subjects were instructed to respond as quickly but also as accurately as possible. When modelling the subjects’ reaction times we must formalize this speed-accuracy trade-off. Let us first define the loss function measuring the cost of *what* (choice $c$) and *when* (reaction time $t$) the subjects decide by:

A1

where we have chosen a squared error loss to encode the estimation loss (the accuracy term) and $h_{\theta}$ encodes the decision delay loss (the speed term), which is assumed to be a strictly monotonically increasing function of within-trial time $t$ (parameterized by $\theta$). This yields the form of the posterior risk as a function of the sufficient statistics $\mu^{(1)}$ of the subject’s posterior belief about the outcome category $x^{(1)}$ (c.f. Robert 1992):

A2

where $\mu^{(1)}\left(t\right)\equiv P\left(x=1\left|u\right.\right)$ is the subject’s posterior representation of the outcome identity at within-trial time $t$ (see below). The gradient of $Q_{\theta}$ w.r.t. within-trial time (i.e. its rate of change ) is thus given by:

A3

Let us define $\tau*$ as the set of times that are local minima for the risk $Q_{\theta}$:

. A4

Given that the delay loss is a monotonically increasing function of time (i.e. ), the set $\tau*$ is not empty ($\tau*\neq\emptyset$) if either of two following conditions are satisfied:

(C1) and $c=1$, or (C2) and $c=0$, which can be rewritten in a more compact form as:

. A5

Equation A5 expresses the necessary condition for the posterior risk $Q_{\theta}$ to possess well-defined (local) minimum points. If Equation A5 is not satisfied, then the risk at the time origin is such that $Q\left(c,0\right)\leq Q\left(c,t\right) \forall t$, and the optimal decision time is zero. In this case, the optimal choice is $c=1$ if and $c=0$ if . This means that the optimal choice depends entirely on the prior prediction $\mu^{(1)}\left(0\right)$, which can potentially lead to a *wrong* choice, i.e. a perceptual categorization error.

It can be seen that equation A2 induces a nonlinear interaction between choices and reaction times, which strongly depends upon the dynamics of both the sufficient statistics $\mu^{(1)}\left(t\right)$ and the delay loss $h_{\theta}\left(t\right)$. In order to allow for an analytical solution to the optimal reaction time in Eq. A4, we must specify the form of the dynamics of both $\mu^{(1)}\left(t\right)$ and $h_{\theta}\left(t\right)$. First, we can motivate the form of $\mu^{(1)}\left(t\right)$ by assuming that the representation performs a gradient ascent on the perceptual free-energy (or negative surprise) $F^{(p)}$. This has been recently suggested as a neurophysiologically plausible implementation of the variational Bayesian approach to perception (Friston et al. 2006, Friston 2008). This process is computationally efficient and uniquely determined if we approximate the free-energy with a quadratic function of its sufficient statistic:

A6

Here, $\mu_{\infty}^{(1)}\equiv\mathrm{argmax}F^{(p)}$ is the optimal value that would be obtained after a very long (infinite) exposure to the stimulus. The parameter $\alpha$ corresponds to the curvature of the perceptual free-energy (and absorbs the unknown rate of ascent, which we have assumed is unity in Equation A6). This curvature is closely related to the posterior precision or confidence about hidden states (see Friston et al., 2007 for details). Equation A5 means that the posterior belief will converge exponentially on its optimal value as time proceeds:

A7

where $\mu_{0}^{(1)}\equiv\mu^{(1)}\left(0\right)$ corresponds to the prior expectation (the prediction from the previous trial) and $\Delta\mu_{0}^{(1)}=\mu_{\infty}^{(1)}-\mu_{0}^{(1)}$ can be thought of as a *post-hoc* prediction error (updated representation minus prior prediction).

This scheme is different from evidence accumulation schemes (e.g. Gold & Shadlen 2001; Glimcher 2003; Carpenter & Williams 1995), whose recognition dynamics are driven "bottom-up" by the attributes of noisy sensory data, e.g. signal-to-noise ratio. In contrast, in our framework, the recognition dynamics are attributed to the process of *inference* and reflect how the brain reorganises its representations in the face of new information. These dynamics are thus the combined result of bottom-up and top-down influences within the hierarchy of the perceptual model.

Substituting recognition dynamics (Eq. A7) into the posterior risk (Eq. A2) allows one to express the posterior risk as a function of *post-hoc* prediction error

$Q_{\theta}\left(c,t\right)=\left(1-2c\right)\mu_{0}^{(1)}+\left(1-2c\right)\Delta\mu_{0}^{(1)}\left(1-\exp\left(-2\alpha t\right)\right)+c+h_{\theta}\left(t\right)$, A8

and yields the following implicit definition of the optimal time for decision and thus reaction time (when comparing this to the main text, note that $\alpha$ in Eq. A9 corresponds to $\theta_{2}$ in Eq. 11):

, A9

Equation A9 cannot be solved explicitly for any delay loss $h_{\theta}\left(t\right)$. However, one can analytically derive the gradients of the optimal reaction time with respect to the parameters of the response model (see equation 9 in the companion paper ‘*Observing the observer (I): meta-Bayesian models of learning and decision making*’):

, A10

where the scaling factor $\varpi$ is such that . Note that for sublinear delay losses $h_{\theta}$ (i.e. losses that increase slower than a linear function of time) the sign of the scaling factor $\varpi\left(t\right)$ can change as a function of time. For example, consider the following delay loss: $h_{\theta}\left(t\right)=t^{\theta}:0<\theta<1$. Then the scaling factor is such that: $\varpi\left(t\right)=0\Leftrightarrow t=\left(1-\theta\right)/2\alpha$ . If this happens in the vicinity of the optimal decision time, then $1/\varpi\left(\tau*\right)\to\infty$ and thus $\tau*$ becomes unstable (an infinitesimal change in the parameters would lead to an infinite change in the optimal reaction time). This is important, since this means that the inversion of the response model would be ill-posed. Thus, as far as response model identification is concerned, linear or superlinear delay losses should be favoured. It turns out that both an exponential loss and a linear loss yield the exact same analytical form for the optimal decision time. This means that when observing the observer, linear and exponential delay losses are not distinguishable from each other (although they are both invertible). We thus choose to use linear delay losses ($h_{\theta}\left(t\right)=\theta t$), for which the optimal decision time is given by:

$\tau*=\frac{1}{2\alpha}\ln\frac{2\alpha\left(2c-1\right)\Delta\mu_{0}^{(1)}}{\theta}$, A11

where $\theta$ is the unknown parameter of the linear delay loss $h_{\theta}\left(t\right)=\theta t$.

Assuming that the representation at the optimal decision time has almost converged (i.e. $\mu^{(1)}\left(\tau*\right)\approx\mu_{\infty}^{(1)}$), it is straightforward to show that the initial posterior risk $Q\left(c,0\right)$ cannot be smaller than the posterior risk at the optimal decision time $Q\left(c,\tau*\right)$, if $\tau*$ exists and is non negative (this happens whenever $\theta$ is small enough). This means that the reaction time $\tau\left(c\right)$ which globally minimizes the posterior risk is:

$\tau\left(c\right)=\left\{\begin{matrix} \tau*\text{ if }\frac{2\alpha\left(2c-1\right)\Delta\mu_{0}^{(1)}}{\theta}>1 \\ 0\text{ otherwise} \end{matrix}\right.$ A12

Equations A11 and A12 form our response model for reaction times, as used in the main text (note that, in terms of notations, we have used $\left[\theta_{1},\theta_{2}\right]\leftarrow\left[\theta,\alpha\right]$).

**References**

Robert C. (1992) *L’analyse statistique Bayesienne*. Economica.

Friston K., Kilner J., Harrison L. (2006) *A free-energy principle for the brain*, J. of physiol. Paris, 100:70-87.

Friston K. (2008), *Hierarchical Models in the Brain*. PLoS Comput Biol 4(11): e1000211.

Friston K., Mattout J., Trujillo-Barreto N., Ashburner J., Penny W. (2007), *Variational free-energy and the Laplace approximation*. NeuroImage 1: 220-234.

Gold JI, Shadlen MN. (2001), *Neural computations that underlie decisions about sensory stimuli*. Trends Cogn. Sci. 5(1): 10-16.

Glimcher PW. (2003), *The neurobiology of visual-saccadic decision making*. Annu Rev. Neurosci. 2003;26:133-79.

Carpenter RH, Williams ML. (1995), *Neural computation of log likelihood in control of saccadic eye movements*. Nature. 377: 59-62.
